# 2-[(4-Bromo­phenyl­imino)­meth­yl]-4,6-di­iodo­phenol

**DOI:** 10.1107/S160053681200551X

**Published:** 2012-02-24

**Authors:** Hao Ji, Hua-Ping Ma, Yong-An Yang, Hai-Liang Zhu

**Affiliations:** a State Key Laboratory of Pharmaceutical Biotechnology, Nanjing University, Nanjing 210093, People’s Republic of China, and Jiangsu Tiansheng Pharmaceutical Company Limited, Jurong Jiangsu 212415, People’s Republic of China

## Abstract

The title compound, C_13_H_8_BrI_2_NO, was prepared by the reaction of 3,5-diiodo­salicyl­aldehyde with 4-bromo­phenyl­amine in ethanol. There is an intra­molecular O—H⋯N hydrogen bond in the mol­ecule, which generates an *S*(6) ring. The dihedral angle between the benzene rings is 2.6 (3)°.

## Related literature

For the biological activities of Schiff bases, see: Chohan *et al.* (2012 ▶); Yan *et al.* (2011 ▶); Zhang *et al.* (2011 ▶). For the coordination of Schiff bases, see: You *et al.* (2008 ▶); Xu *et al.* (2009 ▶); Chen *et al.* (2010 ▶); Cui *et al.* (2011 ▶). For reference bond lengths, see: Allen *et al.* (1987 ▶).
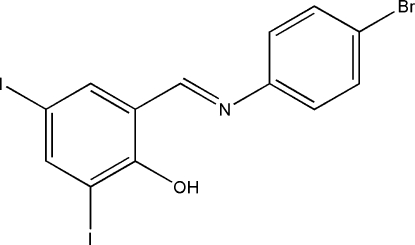


## Experimental

### 

#### Crystal data

C_13_H_8_BrI_2_NO
*M* *_r_* = 527.91
Triclinic, 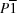

*a* = 7.9870 (13) Å
*b* = 8.9811 (14) Å
*c* = 11.3907 (18) Å
α = 91.093 (2)°
β = 99.873 (2)°
γ = 114.570 (2)°
*V* = 728.4 (2) Å^3^
*Z* = 2
Mo *K*α radiation
μ = 7.05 mm^−1^
*T* = 298 K
0.17 × 0.15 × 0.15 mm

#### Data collection

Bruker SMART CCD area-detector diffractometer
Absorption correction: multi-scan (*SADABS*; Sheldrick, 1996 ▶) *T*_min_ = 0.380, *T*_max_ = 0.418
6174 measured reflections
3125 independent reflections
2425 reflections with *I* > 2σ(*I*)
*R*_int_ = 0.022

#### Refinement

*R*[*F* ^2^ > 2σ(*F* ^2^)] = 0.032
*wR*(*F* ^2^) = 0.094
*S* = 1.07
3125 reflections
164 parameters
H-atom parameters constrained
Δρ_max_ = 1.26 e Å^−3^
Δρ_min_ = −0.76 e Å^−3^

### 

Data collection: *SMART* (Bruker, 1998 ▶); cell refinement: *SAINT* (Bruker, 1998 ▶); data reduction: *SAINT*; program(s) used to solve structure: *SHELXS97* (Sheldrick, 2008 ▶); program(s) used to refine structure: *SHELXL97* (Sheldrick, 2008 ▶); molecular graphics: *SHELXTL* (Sheldrick, 2008 ▶); software used to prepare material for publication: *SHELXTL* .

## Supplementary Material

Crystal structure: contains datablock(s) global, I. DOI: 10.1107/S160053681200551X/qm2053sup1.cif

Structure factors: contains datablock(s) I. DOI: 10.1107/S160053681200551X/qm2053Isup2.hkl

Supplementary material file. DOI: 10.1107/S160053681200551X/qm2053Isup3.cml

Additional supplementary materials:  crystallographic information; 3D view; checkCIF report

## Figures and Tables

**Table 1:** Hydrogen-bond geometry (Å, °)

*D*—H⋯*A*	*D*—H	H⋯*A*	*D*⋯*A*	*D*—H⋯*A*
O1—H1⋯N1	0.82	1.85	2.576 (5)	148

**Fig. 1.**
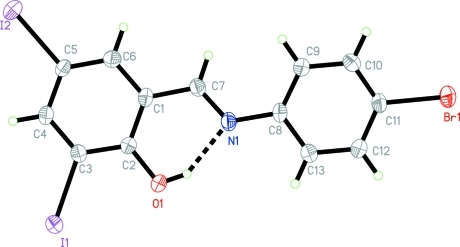
The molecular structure of the title compound, showing the atom labelling scheme. The displacement ellipsoids are drawn at the 30% probability level. The intramolecular hydrogen bond is indicated by a dashed line.

## supplementary crystallographic information

### Comment

Schiff bases have been extensively studied for their biological activities (Chohan *et al.*, 2012; Yan *et al.*, 2011; Zhang *et al.*, 2011). In addition, Schiff bases are versatile ligands for the preparation of metal complexes (You *et al.*, 2008; Xu *et al.*, 2009; Chen *et al.*, 2010; Cui *et al.*, 2011). In the present paper, the new title compound is reported.

The molecule of the compound exists in a *trans* configuration with respect to the methylidene unit (Fig. 1). There is an intramolecular O1—H1···N1 hydrogen bond in the molecule (Table 1). The dihedral angle between the C1–C6 and C8–C13 benzene rings is 2.6 (3)°. The bond distances are within the normal range (Allen *et al.*, 1987).

### Experimental

3,5-Diiodosalicylaldehyde (0.37 g, 1 mmol) and 4-bromophenylamine (0.17 g, 1 mmol) were mixed in ethanol (20 ml). The mixture was stirred at room temperature for 30 min to give a yellow solution. Yellow block-shaped single crystals were obtained by slow evaporation of the solution in air.

### Refinement

H-atoms were positioned geometrically and refined using a riding model, with C—H = 0.93–0.97 Å, O—H = 0.82 Å, and with *U*_iso_(H) set to 1.2*U*_eq_(C) and 1.5*U*_eq_(O).

### Crystal data

**Table:**

C_13_H_8_BrI_2_NO	*Z* = 2
*M**_r_* = 527.91	*F*(000) = 484
Triclinic, *P*1	*D*_x_ = 2.407 Mg m^−^^3^
*a* = 7.9870 (13) Å	Mo *K*α radiation, λ = 0.71073 Å
*b* = 8.9811 (14) Å	Cell parameters from 1027 reflections
*c* = 11.3907 (18) Å	θ = 2.5–25.1°
α = 91.093 (2)°	µ = 7.05 mm^−^^1^
β = 99.873 (2)°	*T* = 298 K
γ = 114.570 (2)°	Block, yellow
*V* = 728.4 (2) Å^3^	0.17 × 0.15 × 0.15 mm

### Data collection

**Table:**

Bruker SMART CCD area-detector diffractometer	3125 independent reflections
Radiation source: fine-focus sealed tube	2425 reflections with *I* > 2σ(*I*)
Graphite monochromator	*R*_int_ = 0.022
ω scans	θ_max_ = 27.0°, θ_min_ = 1.8°
Absorption correction: multi-scan (*SADABS*; Sheldrick, 1996)	*h* = −10→10
*T*_min_ = 0.380, *T*_max_ = 0.418	*k* = −10→11
6174 measured reflections	*l* = −14→14

### Refinement

**Table:**

Refinement on *F*^2^	Primary atom site location: structure-invariant direct methods
Least-squares matrix: full	Secondary atom site location: difference Fourier map
*R*[*F*^2^ > 2σ(*F*^2^)] = 0.032	Hydrogen site location: inferred from neighbouring sites
*wR*(*F*^2^) = 0.094	H-atom parameters constrained
*S* = 1.07	*w* = 1/[σ^2^(*F*_o_^2^) + (0.0399*P*)^2^ + 1.2928*P*] where *P* = (*F*_o_^2^ + 2*F*_c_^2^)/3
3125 reflections	(Δ/σ)_max_ < 0.001
164 parameters	Δρ_max_ = 1.26 e Å^−^^3^
0 restraints	Δρ_min_ = −0.76 e Å^−^^3^

### Special details

**Table:**

Geometry. All e.s.d.'s (except the e.s.d. in the dihedral angle between two l.s. planes) are estimated using the full covariance matrix. The cell e.s.d.'s are taken into account individually in the estimation of e.s.d.'s in distances, angles and torsion angles; correlations between e.s.d.'s in cell parameters are only used when they are defined by crystal symmetry. An approximate (isotropic) treatment of cell e.s.d.'s is used for estimating e.s.d.'s involving l.s. planes.
Refinement. Refinement of *F*^2^ against ALL reflections. The weighted *R*-factor *wR* and goodness of fit *S* are based on *F*^2^, conventional *R*-factors *R* are based on *F*, with *F* set to zero for negative *F*^2^. The threshold expression of *F*^2^ > σ(*F*^2^) is used only for calculating *R*-factors(gt) *etc*. and is not relevant to the choice of reflections for refinement. *R*-factors based on *F*^2^ are statistically about twice as large as those based on *F*, and *R*- factors based on ALL data will be even larger.

### Fractional atomic coordinates and isotropic or equivalent isotropic displacement parameters (Å^2^)

**Table:**

	*x*	*y*	*z*	*U*_iso_*/*U*_eq_	
I1	0.87298 (7)	0.30448 (5)	−0.38474 (3)	0.06516 (15)	
Br1	0.27660 (10)	0.16630 (8)	0.47166 (5)	0.0712 (2)	
I2	1.20768 (6)	1.02378 (4)	−0.20734 (4)	0.06502 (15)	
N1	0.6508 (5)	0.3888 (5)	0.0440 (3)	0.0403 (9)	
O1	0.7124 (6)	0.2911 (4)	−0.1515 (3)	0.0532 (9)	
H1	0.6635	0.2839	−0.0929	0.080*	
C1	0.8487 (7)	0.5748 (6)	−0.0729 (4)	0.0390 (10)	
C2	0.8205 (6)	0.4496 (6)	−0.1604 (4)	0.0380 (10)	
C3	0.9082 (7)	0.4926 (6)	−0.2583 (4)	0.0421 (11)	
C4	1.0185 (7)	0.6550 (6)	−0.2713 (4)	0.0444 (11)	
H4	1.0764	0.6820	−0.3371	0.053*	
C5	1.0422 (7)	0.7773 (6)	−0.1854 (4)	0.0418 (11)	
C6	0.9621 (7)	0.7389 (6)	−0.0865 (4)	0.0416 (11)	
H6	0.9830	0.8220	−0.0282	0.050*	
C7	0.7600 (7)	0.5360 (6)	0.0313 (4)	0.0419 (11)	
H7	0.7836	0.6202	0.0894	0.050*	
C8	0.5660 (6)	0.3466 (6)	0.1464 (4)	0.0397 (10)	
C9	0.5782 (8)	0.4581 (7)	0.2364 (5)	0.0554 (14)	
H9	0.6453	0.5703	0.2325	0.066*	
C10	0.4912 (8)	0.4041 (7)	0.3323 (5)	0.0548 (14)	
H10	0.4986	0.4796	0.3922	0.066*	
C11	0.3947 (7)	0.2400 (7)	0.3386 (4)	0.0469 (12)	
C12	0.3773 (8)	0.1262 (7)	0.2497 (5)	0.0547 (14)	
H12	0.3098	0.0143	0.2545	0.066*	
C13	0.4618 (8)	0.1808 (7)	0.1527 (5)	0.0517 (13)	
H13	0.4484	0.1048	0.0910	0.062*	

### Atomic displacement parameters (Å^2^)

**Table:**

	*U*^11^	*U*^22^	*U*^33^	*U*^12^	*U*^13^	*U*^23^
I1	0.0980 (3)	0.0439 (2)	0.0485 (2)	0.0182 (2)	0.0335 (2)	0.00210 (15)
Br1	0.0904 (5)	0.0621 (4)	0.0500 (3)	0.0110 (3)	0.0415 (3)	0.0074 (3)
I2	0.0833 (3)	0.0366 (2)	0.0657 (3)	0.01060 (18)	0.0292 (2)	0.01196 (16)
N1	0.041 (2)	0.044 (2)	0.039 (2)	0.0172 (18)	0.0162 (17)	0.0086 (17)
O1	0.067 (2)	0.0355 (18)	0.047 (2)	0.0065 (16)	0.0266 (18)	0.0060 (15)
C1	0.041 (3)	0.042 (3)	0.037 (2)	0.018 (2)	0.0134 (19)	0.0135 (19)
C2	0.039 (2)	0.036 (2)	0.038 (2)	0.0129 (19)	0.0105 (19)	0.0054 (18)
C3	0.049 (3)	0.044 (3)	0.036 (2)	0.020 (2)	0.013 (2)	0.007 (2)
C4	0.053 (3)	0.043 (3)	0.040 (2)	0.019 (2)	0.018 (2)	0.013 (2)
C5	0.047 (3)	0.032 (2)	0.046 (3)	0.013 (2)	0.018 (2)	0.012 (2)
C6	0.044 (3)	0.040 (3)	0.043 (2)	0.019 (2)	0.010 (2)	0.004 (2)
C7	0.049 (3)	0.043 (3)	0.040 (2)	0.022 (2)	0.017 (2)	0.008 (2)
C8	0.037 (2)	0.047 (3)	0.038 (2)	0.017 (2)	0.0153 (19)	0.010 (2)
C9	0.070 (4)	0.041 (3)	0.049 (3)	0.012 (3)	0.028 (3)	0.005 (2)
C10	0.070 (4)	0.047 (3)	0.042 (3)	0.016 (3)	0.022 (3)	−0.002 (2)
C11	0.049 (3)	0.051 (3)	0.038 (2)	0.015 (2)	0.019 (2)	0.007 (2)
C12	0.063 (3)	0.043 (3)	0.053 (3)	0.011 (2)	0.027 (3)	0.007 (2)
C13	0.063 (3)	0.045 (3)	0.049 (3)	0.020 (3)	0.026 (3)	0.005 (2)

### Geometric parameters (Å, º)

**Table:**

I1—C3	2.093 (5)	C5—C6	1.369 (7)
Br1—C11	1.907 (5)	C6—H6	0.9300
I2—C5	2.101 (5)	C7—H7	0.9300
N1—C7	1.273 (6)	C8—C9	1.382 (7)
N1—C8	1.427 (6)	C8—C13	1.382 (7)
O1—C2	1.340 (6)	C9—C10	1.382 (7)
O1—H1	0.8200	C9—H9	0.9300
C1—C6	1.401 (7)	C10—C11	1.360 (8)
C1—C2	1.406 (7)	C10—H10	0.9300
C1—C7	1.460 (6)	C11—C12	1.373 (7)
C2—C3	1.394 (6)	C12—C13	1.385 (7)
C3—C4	1.382 (7)	C12—H12	0.9300
C4—C5	1.387 (7)	C13—H13	0.9300
C4—H4	0.9300		
			
C7—N1—C8	122.4 (4)	N1—C7—H7	119.4
C2—O1—H1	109.5	C1—C7—H7	119.4
C6—C1—C2	119.6 (4)	C9—C8—C13	118.8 (4)
C6—C1—C7	119.6 (4)	C9—C8—N1	125.0 (5)
C2—C1—C7	120.8 (4)	C13—C8—N1	116.1 (4)
O1—C2—C3	119.6 (4)	C8—C9—C10	120.4 (5)
O1—C2—C1	121.7 (4)	C8—C9—H9	119.8
C3—C2—C1	118.7 (4)	C10—C9—H9	119.8
C4—C3—C2	121.2 (4)	C11—C10—C9	119.7 (5)
C4—C3—I1	120.4 (3)	C11—C10—H10	120.1
C2—C3—I1	118.4 (4)	C9—C10—H10	120.1
C3—C4—C5	119.3 (4)	C10—C11—C12	121.3 (5)
C3—C4—H4	120.3	C10—C11—Br1	119.5 (4)
C5—C4—H4	120.3	C12—C11—Br1	119.2 (4)
C6—C5—C4	121.0 (4)	C11—C12—C13	118.9 (5)
C6—C5—I2	120.1 (4)	C11—C12—H12	120.6
C4—C5—I2	118.9 (3)	C13—C12—H12	120.6
C5—C6—C1	120.2 (4)	C8—C13—C12	120.8 (5)
C5—C6—H6	119.9	C8—C13—H13	119.6
C1—C6—H6	119.9	C12—C13—H13	119.6
N1—C7—C1	121.2 (4)		

### Hydrogen-bond geometry (Å, º)

**Table:**

*D*—H···*A*	*D*—H	H···*A*	*D*···*A*	*D*—H···*A*
O1—H1···N1	0.82	1.85	2.576 (5)	148
